# The cold truth: torpor as a confound in studies of caloric restriction

**DOI:** 10.1007/s00360-025-01616-1

**Published:** 2025-06-09

**Authors:** William S. R. Wheatley, Christopher J. Marshall, Ludovico Taddei, Timna Hitrec, Anthony E. Pickering, Michael T. Ambler

**Affiliations:** 1 School of Physiology, Pharmacology & Neuroscience, University of Bristol, Bristol, BS8 1TD UK; 2 Department of Biomedical and Neuromotor Sciences, University of Bologna, Bologna, 40127 Italy

**Keywords:** Calorie restriction, Fasting, Torpor, Longevity, Health, Diet

## Abstract

Calorie restriction has been shown to dramatically extend lifespan in a range of species. Beyond longevity, calorie restriction is also reported to improve cognitive function, ameliorate neurodegeneration and peripheral nerve damage, reduce cancer incidence, and is commonly used to increase motivation in studies of behaviour. The mouse has been the most common species for these experiments and whilst efforts are ongoing to demonstrate the benefits of calorie restriction in humans, the evidence in mice is most compelling. Many mechanisms have been proposed for the beneficial effects of calorie restriction, but we note that one potentially important factor has seldom been considered: namely that mice readily enter torpor in response to food restriction. Torpor is a remarkable protective physiological state characterized by profound reductions in body temperature, oxygen consumption, heart rate, and activity. In this review, we describe the dietary protocols used to study the effects of calorie restriction and present the case that mice in these studies are highly likely to have entered torpor. We discuss the extent to which torpor might influence or mediate the measured outcomes. We highlight that induction of torpor is an important confound that is rarely, if at all, considered in calorie restriction research and make recommendations for the design and conduct of future studies.

**Supplementary Information:**

The online version contains supplementary material available at 10.1007/s00360-025-01616-1.

## Introduction

**Fig. 1:**
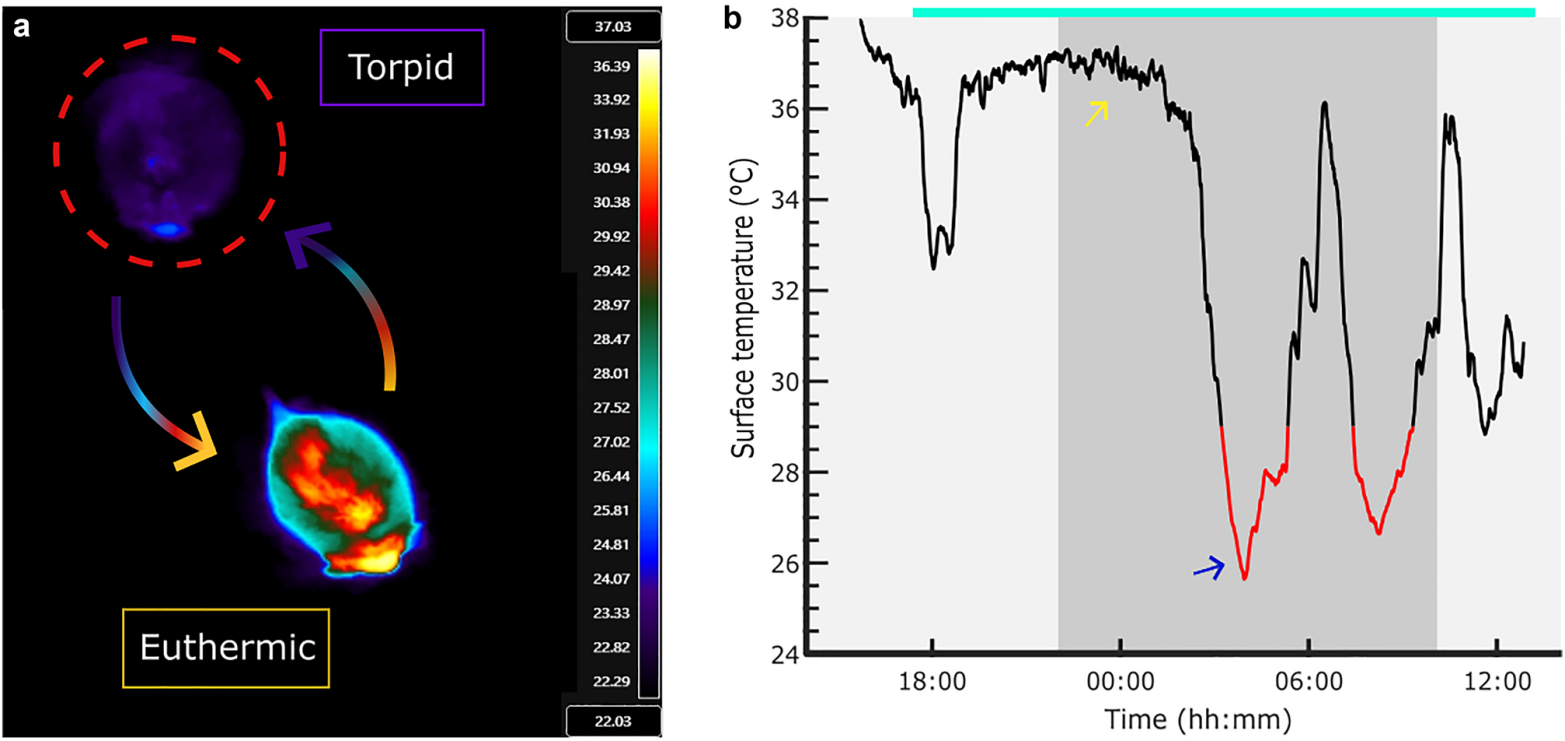
Example thermal image and surface temperature trace of a mouse during a 24-h fast. **a** A thermal image of the same mouse whilst in active euthermia and while torpid (dashed red circle) during a 24-h fast. Temperature is in Celsius. **b** A trace of surface temperature during the same 24-h fast. Food was removed from 5:00pm (cyan line), 5 h before lights-off (10:00pm). The red lines correspond to heterothermic bouts identified by our criteria that maximum surface temperature must fall below 29°C for at least 30 min. The yellow and blue arrows correspond to the timepoints for the euthermic and torpid images from **a,** respectively

Torpor is an adaptive, protective physiological state, engaged by a range of mammals and birds, typically in response to environmental challenge such as a drop in ambient temperature, reduced photoperiod, or a reduction in food availability (Lyman et al. 1982). Key physiological features of torpor are a dramatic drop in metabolic rate (Hudson 1979; Heldmaier et al. 2004; Brown and Staples 2010), a controlled reduction in core body temperature (Fig. 1), (Hudson 1979), and a dramatically slowed heart rate (Heldmaier et al. 2004). Torpor enables animals to tolerate food scarcity and environmental hardship, conferring a survival advantage over homeothermic counterparts in the wild (Geiser and Turbill 2009; Turbill et al. 2011; Turbill and Stojanovski 2018).

Torpor duration ranges from several hours in daily heterotherms such as mice, to several days in seasonal hibernators such as ground squirrels. The triggers for torpor also vary from species to species. Mice will enter daily torpor when the energy demands of maintaining normothermia exceed what can be delivered by food intake. Most commonly, this would occur when food availability is compromised, but daily torpor can also be triggered in mice when the energy demands of obtaining food are high (Schubert et al. 2010). On the other hand, obligate seasonal hibernators enter prolonged periods of torpor in response to the changing season, usually following a period of high calorie intake. Finally, facultative hibernators will enter either daily or prolonged torpor bouts (depending on the species) in response to reduced ambient temperature or shortened photoperiod (Janský et al. 1984) (see (Ruf and Geiser 2015) for a review of differences between species that engage daily or extended periods of torpor).

Understanding of the precise endogenous signals that trigger and control torpor is incomplete, but recent pioneering studies have shown the preoptic area of the hypothalamus is a key locus, capable of recapitulating key features of torpor (reduced oxygen consumption, reduced body temperature, and bradycardia) (Hrvatin et al. 2020; Takahashi et al. 2020; Zhang et al. 2020; Ambler et al. 2022). Circulating hormones such as leptin (Gavrilova et al. 1999; Freeman et al. 2004) and ghrelin (Sato et al. 2021) are also likely to play a role in triggering torpor.

Particularly in the case of hibernators, torpor is associated with several beneficial physiological adaptations beyond simply reduced oxygen and nutrient consumption. These include changes in telomere dynamics (Turbill et al. 2012), increased tolerance to ischaemia–reperfusion injury (Zancanaro et al. 1999; Jani et al. 2011), and avoidance of sarcopenia that one might expect from prolonged periods of inactivity or starvation (Gao et al. 2012). Additionally, housing Turkish hamsters (*Mesocriceus brandti*) under cold ambient temperatures (5°C + −2°C) results in regular torpor bouts lasting 3–6 days (Batavia et al. 2013) and is associated with a greater than 10% increase in longevity compared to warm-housed controls that did not engage torpor (Lyman et al. 1981).

At the same time, there is extensive literature describing the effects of long term caloric restriction and/or periods of fasting in mice, including: delayed aging-related physiological deterioration (Mitchell et al. 2016; Xie et al. 2020; Green et al. 2022) and extended longevity (Weindruch et al. 1986; Mitchell et al. 2019; Green et al. 2022), improved glucose and insulin regulation (Anson et al. 2003), increased neurogenesis (Lee et al. 2002; Baik et al. 2020), improved cognitive performance (Bang et al. 2022), reduced cancer incidence (Lee et al. 2012; Mattison et al. 2012), reversed deficits from neurodegenerative disorders like Alzheimer’s (Halagappa et al. 2007; Elias et al. 2023) and Parkinson’s disease (Maswood et al. 2004), improved peripheral nerve repair (Serger et al. 2022), and reduced damage following traumatic brain injury (Cao et al. 2022; Yang et al. 2023) and spinal cord injury (Plunet et al. 2008). These studies, while not explicitly setting out to study torpor, use calorie restriction protocols that we believe are highly likely to induce torpor in the mice.

In this review, we characterise the dietary protocols that are widely used to induce torpor in mice and compare these to protocols employed in the calorie restriction literature to establish the likelihood of unwittingly inducing torpor. We then explore how torpor may have influenced the results and discuss what steps could be taken to determine whether results are attributable to the dietary alteration alone, rather than the engagement of torpor.

## Calorie restriction protocols designed to induce torpor

**Fig. 2:**
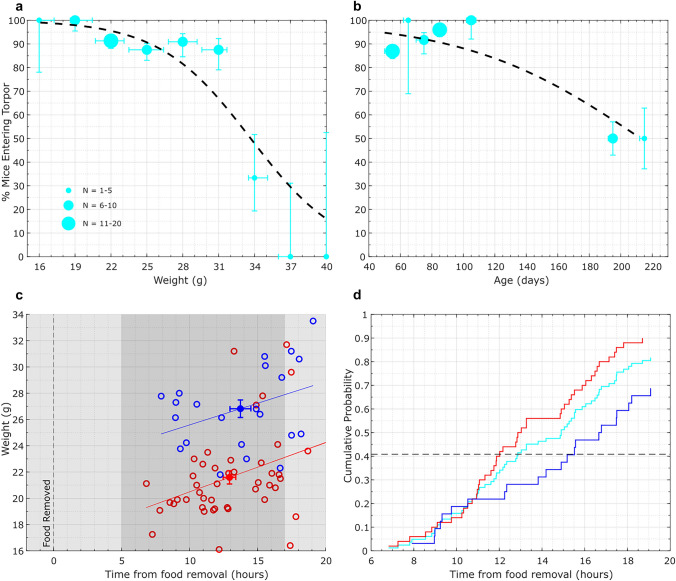
Data from 24-h fast protocols across experiments performed in our lab. Data combined from experiments measuring surface temperature (N = 63) or core temperature (N = 20). **a & b** Male and female mice (N = 83) aggregated into 3g weight bins (**a**) or grouped by 10-day age bins (**b**). Vertical error bars show binomial confidence interval and horizontal error bars show the range of weights (**a**) or ages (**b**) contained within each band. A logistic regression model (dashed black line) has been fitted to the data. The key shows the number of mice in each bin denoted by point size. **c** A scatter plot showing torpor latency for male (blue) and female (red) mice—the time taken from food removal to torpor entry plotted against their weight at the time of food removal. Mice that did not enter torpor (n = 11) or where latency data was not available (n = 6) are not included. The light and dark grey backgrounds correspond to lights-on and lights-off respectively. The dashed black vertical line corresponds to the time when food was removed. The filled circles and error bars represent mean and standard error respectively. Pairwise linear correlations are shown for the male (y = 0.33x + 22.28) and female (y = 0.36x + 16.77) groups separately. Only the females show a significant correlation between weight and torpor latency (correlation coefficient = 0.3752, p = 0.0111). There was no significant difference in time to enter torpor between males (mean = 13.7, SD = 3.60) and females (mean = 12.9, SD = 3.09), t(64) = −0.98, p = 0.33.). **d—**Cumulative probability plot for the data from female mice (red), male mice (blue), and both combined (cyan). Only data from mice without torpor latency data were excluded (leaving 77 mice in this plot). All animals that entered torpor (66/77) had done so within 20 h and half of these had entered torpor within 13 h (dashed black line). As time from food removal increases, each additional animal that enters torpor increases the cumulative probability by—total % that entered torpor/total number of animals

Complete fasting periods (with all food removed) in mouse torpor studies can last up to 72 h (Brown and Staples 2010; Solymár et al. 2015) during which several torpor bouts are commonly observed (Brown and Staples 2010). Studies that employ a shorter 24-h fasting period consistently report torpor entry (Swoap et al. 2007; Swoap and Gutilla 2009; Sunagawa and Takahashi 2016; Lo Martire et al. 2018). Indeed, fasting for as little as 12-h can be sufficient to induce torpor provided the fast is initiated at the onset of the “active phase” lights-off period (Swoap et al. 2007; Swoap and Gutilla 2009; Lo Martire et al. 2018; Hrvatin et al. 2020) and not at the onset of the “resting phase” lights-on period (Mitchell et al. 2015; Sunagawa and Takahashi 2016). This reflects the circadian partitioning of food intake and activity, and the circadian gating of torpor (Heller and Ruby 2004) (Fig. 2c). The likelihood of torpor entry can be affected by several factors beyond the dietary intervention: torpor is more frequently observed in cooler ambient temperatures (21°C and below, compared to 28°C Hitrec et al. 2019; Hudson 1979)), is more likely if the mice are lighter in weight (Mitchell et al. 2015; Solymár et al. 2015), if the mice are female (Swoap and Gutilla 2009; Mitchell et al. 2016), and if eating requires greater energy expenditure (Schubert et al. 2010). Certain genetic strains also show greater propensity to enter torpor (Rikke et al. 2003).

In addition to periods of complete food deprivation, single daily meal feeding that enforces a degree of calorie restriction can induce torpor (Mitchell et al. 2015; Van Der Vinne et al. 2018; Ambler et al. 2022). For example, when mice receive approximately 70% of their ad libitum intake in a single daily meal, torpor is observed after 2 or 3 days (Brown and Staples 2010; Huang et al. 2021; Ambler et al. 2022). A 30% reduction in food intake appears to be a threshold when single-meal feeding since both 20% (Mitchell et al. 2015) and 25% (Guijas et al. 2020) less than ad libitum does not trigger torpor.

### Defining torpor

Criteria for identifying torpor vary and are inevitably somewhat arbitrary. Reduced metabolic rate is both the initial and the defining feature of torpor (Tøien et al. 2011). However, measuring oxygen consumption is costly and technically challenging and so studies typically focus on a prolonged and pronounced reduction in core or surface body temperature (Brown and Staples 2010). Our group uses a core temperature threshold of 33°C and a surface temperature threshold of 29°C (with the added requirement that surface temperature remained below 29°C for at least 30 min). These criteria are adapted from Ambler et al. (2022), with 29°C as an approximation for 4 standard deviations below the typical surface temperature mean. We deliberately use conservative criteria for torpor (Ambler et al. 2022) to avoid ascribing ordinary fluctuations in body temperature to torpor. Less strict criteria would potentially identify torpor earlier or more frequently, but at the cost of specificity (Fig. 1b).

### Likelihood of torpor following 24-h fast

To quantify the likelihood of mice entering torpor during a 24-h fast, we analysed unpublished datasets from experiments performed by our group (Fig. 2). These data are predominantly surface temperature thermal using thermal cameras (Flir C2). However, 20 animals were implanted with intraperitoneal telemetric probes (AnipillV2, BodyCAP, Hérouville Saint Clair, France). All mice are from a C57BL/6J background, though some are transgenic (TRAP2 (https://www.jax.org/strain/030323) or Adcyap1-CRE lines (https://www.jax.org/strain/033999)). Mice were housed on a 12h:12h light–dark cycle (light 10:00–22:00), at 21 + −1°C with ad libitum access to food (EUROdent Diet 22%, irradiated, 5LF5) and water. On fast days, mice were transported to a custom-built cage and food was removed at 1700 +−1 h.

Across the whole population, the starting weight (Fig. 2a) and/or age (Fig. 2b) of the mouse affects the likelihood of torpor, with heavier and older mice less likely to enter torpor. Mice below 30 g had a > 90% chance of entering torpor (n = 70), whilst only 54% of mice weighing 30g or more entered torpor (n = 13). When examined in terms of age, mice under 4 months at the time of food removal had a 93% chance of entering torpor (n = 71) but those over 4 months had only a 50% chance of entering torpor (n = 12). From our data it is unclear whether age or weight is the dominant determinant for torpor entry. However, 6-month-old obese mice (> 50 g) fasted for a week do not enter torpor, whilst 24g age-matched controls all entered torpor within three days of fasting (Solymár et al. 2015). This suggests that body mass might be the critical factor, at least up to 6 months of age. Observing torpor propensity in aged mice (over 1 year) could help distinguish the degree to which age is a factor because beyond 1 year weight is more stable than in younger mice (Evans et al. 2021).

## Calorie restriction protocols used outside of torpor studies

The precise feeding protocols used to explore the effects of calorie restriction in mice vary from study to study, but they often follow closely, or even identically, the protocols used to induce torpor. Commonly used protocols employ a single fast period (Pietrocola et al. 2017; Nakamura et al. 2023), an alternate day fasting protocol (also called every other day fast – repeated 24-h ad libitum food availability followed by 24-h fast) (Xie et al. 2017; Pan et al. 2022), or daily provision of a calorie restricted single-meal (Weindruch et al. 1986; Kubo et al. 1992). Single-meal feeding protocols expose mice to both a reduction in total calorie allowance, and a daily fast period of around 21-h due to the normal habit of laboratory mice to ‘meal-gorge’ (Mitchell et al. 2019; Green et al. 2022). This fast period is sufficient for mice to enter torpor (Fig. 2c and d), and indeed 5 days of single-meal feeding with a 30% calorie restriction is sufficient to induce torpor in 97% of trials (Ambler et al. 2022).

Unfortunately, these studies rarely report body temperature, oxygen consumption, or heart rate. These data would allow us to understand whether, or to what extent, the effects of torpor might influence their findings. For this review we searched for papers using the key terms ‘calorie restriction’, ‘longevity’, ‘cognitive function’, ‘immune function’, and ‘mouse behavioural testing’. Table 1 lists only the calorie restriction papers from our search that provide weight information for their mice. This table outlines the strain, sex, weight, age, and ambient temperature, where available, in these studies’ calorie restricted groups, and gives our prediction for the likelihood of those mice entering torpor.

**Table 1:** Table of experiments that implement either 24-h fasts, or single-meal feeding with caloric restriction

Author	Year	Field	Strain	Sex	Ta (°C)	Weight (g)	Age (days)	Predicted Torpor Likelihood (%)	
								Weight	Age
*Acosta-Rodrigue*z et al	2022	Longevity	C57BL/6J	M	–	26	**540**	88.2	**0.3**
*Anson *et al	2003	Longevity	C57BL/6	M	**–**	**15.5** 18.5	133	**99.2** 98.2	80.5
*Cao *et al	2022	Brain injury & neurogenesis	C57BL/6N	M & F	22–25	23	63	94.3	93.5
*Carlini *et al	2008	Memory	SWR/J	F	18–22	25	–	90.7	–
*Chowdhury *et al	2013	Anorexia	C57BL/6	F F	–	16.5 18.5	**38**	98.9 98.2	**95.7**
*Gardner *et al	2005	Immune function	C57BL/6, G42, GIN	M	–	28	**690**	81.6	**0.02**
*Grissom *et al	2018	Autism & learning	B6129SF1/J B6129S-Del	M F	–	22.5 28	70	94.9 81.6	92.7
*Higa *et al	2017	Dopamine & learning	C57BL/6J	M	–	27.5	165	83.5	70.1
*Heinz *et al	2021	Anxiety	CD-1	M	–	30	120	72.4	83.9
*Ineichen *et al	2012	Learning & serotonin	C57BL/6J	M	20–22	23	70	94.3	92.7
*Ingram *et al	1987	Learning & motor skills	C3B10RF	F F	20–24	21	195 **495**	96.5	57.8 **0.7**
*Kubo *et al	1992	Autoimmune disease	BXSB	F M	–	19 20	150	97.9 97.3	75.3
*Lee *et al	2002	Cancer	BDNF ±	M M	20–23	26.5 31	150	86.8 66.9	75.3
*Li *et al	2013	Cognitive function	CD-1	M	–	**43.5**	210	**0.07**	51.2
*Pak *et al	2021	Longevity	C57BL/6J	M	20–22	19 21.5	126	97.9 96.1	82.4
*Pan *et al	2022	Alzheimer’s disease	5XFAD	M	22	22.5	–	94.9	–
*Rangan *et al	2022	Alzheimer’s disease	3xTg E4FAD	F M	–	23 30.5	**315**	94.3 69.7	**14**
*Serger *et al	2022	Nerve repair	C57BL/6	M	20–24	25	**49**	90.7	**94.8**
*Sun *et al	2001	Immune function	C57BL/6	F	24	16.5	120	98.9	83.9
*Sun *et al	2004	Autoimmune disease	NZB/NZW F1	F	–	28	149	81.6	75.7
*Weindruch and Walford*	1982	Longevity & cancer	BlOC3FI	M	–	24.5 28.5	**600**	91.7 79.5	**0.1**
*Weindruch *et al	1986	Longevity & cancer	C3B10RF	F	20–24	19.5	**600**	97.6	**0.1**
*Xie *et al	2017	Longevity	C57BL/6J	M	22	27.5 32.5	90 **500**	83.5 57.7	89.9 **0.6**
*Yang *et al	2014	Cognitive function	C57BL/6J	M	–	27 29.5	**225** **345**	85.2 74.9	**44.6** **8.7**

Papers discussed in this review that did not provide weight information are not included in this table. For long-term experiments, the weight given in the table is representative across the length of the dietary intervention. Otherwise, this weight represents the weight at the start of the dietary intervention or, in the case of behavioural studies, at the start of experiments. If the study includes more than one experimental group, values for both groups ages and weights are given. The final two columns contain our predictions of the likelihood that torpor occurred in these food restricted samples calculated inputting either weight (column 7) or age (column 8) into the logistic regressions from Fig. 2. Two of the experimental group weights and several ages were outside our tested range. These values and their corresponding torpor probabilities are in bold. Ta (°C) refers to ambient temperature

### Predicting torpor likelihood in calorie restriction studies

Logistic regression models were fitted to our collated dataset in MATLAB using the fitglm function (Fig. 2a&b dashed black line). A sigmoid function was used to transform the weight regression parameters (Fig. 2a) from our data with the weight values from Table 1 experiments, outputting the probability of that sample entering torpor (Table 1–“Predicted Torpor Likelihood (%)–weight” column). We then averaged the weight predictions for all Table 1 experiments to give an overall torpor likelihood of 85.8% (standard error = 3.17). This was repeated using the age regression parameters and experiment values and this gave a lower torpor likelihood prediction of 54.6% (standard error = 7.7).

We had concerns our age data was not sufficiently representative (11 Table 1 experiments had animals older or younger than the age range we have tested, and these skewed predictions downwards–removing them increased the mean predicted probability of entering torpor from 54.6% to 78.9%) and therefore use predictions based on our weight logistic regression as the basis for our proposal that > 80% of the animals in experimental groups in these studies are entering torpor each night.

Whilst other factors do impact torpor likelihood, for the experiments listed in Table 1 we are confident that our predictions are credible as the majority of studies were performed at similar ambient temperatures to ours, on a C57BL/6 background, and with similarly low energetic demands beyond ordinary cage exploration.

### Persistent daily torpor over extended periods

**Fig. 3:**
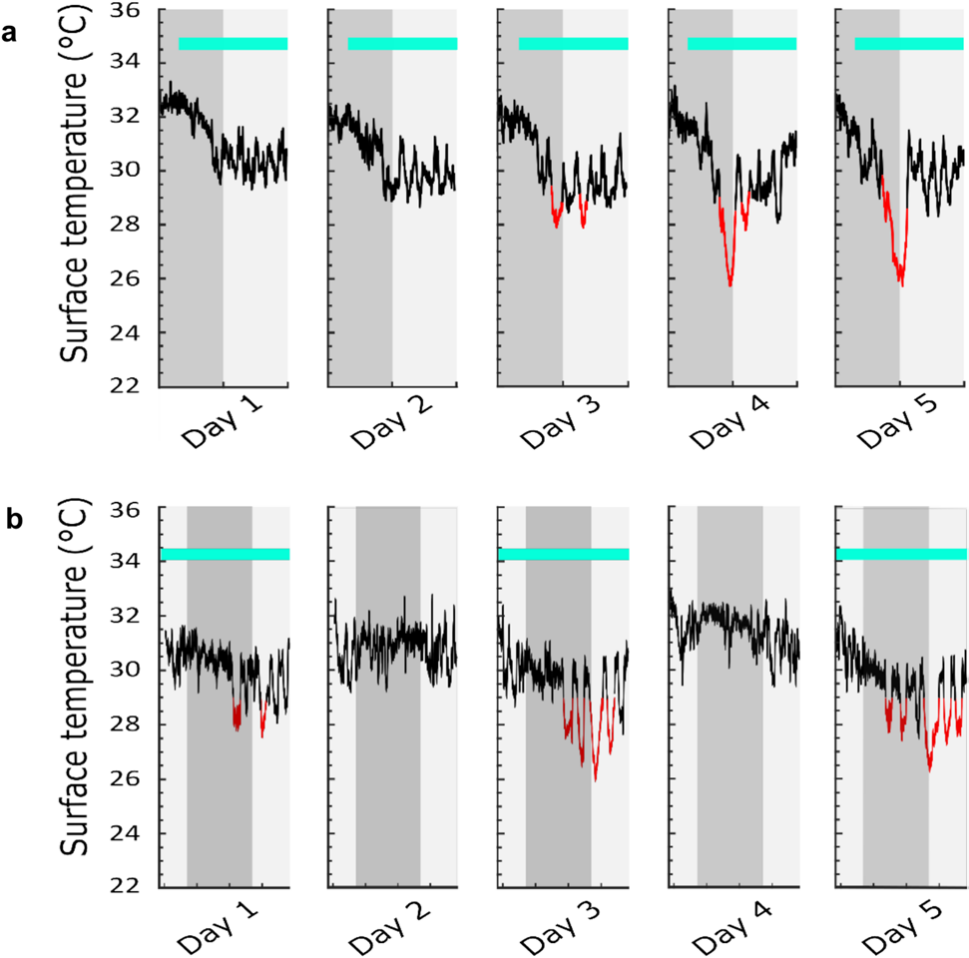
Torpor induction by different feeding protocols – fast periods are indicated by cyan boxes. **a** Surface temperature (black line) from a 25 g female mouse on a 30% caloric restriction provided through a single meal at the start of lights-off. The red lines indicate periods in torpor. These torpor bouts increased in depth and duration as food restriction was continued. (Ambler et al 2022). **b** Surface temperature (black line) from a 31 g female mouse receiving alternating 24-h periods of ad-libitum feeding (cyan box) or fasting. The red lines indicate periods in torpor that continued to occur through the fast/feed cycles

On a short-term basis, the likelihood of torpor increases with the duration of the calorie restriction. For example, when fasted for 72 h (Brown and Staples 2010) or on a single-meal feeding protocol lasting several days (Van Der Vinne et al. 2018; Ambler et al. 2022), mice exhibit an increasing probability of torpor with progressively deeper and longer bouts each day (Fig. 3a). However, studies that employ calorie restriction to investigate the effects on health and longevity often last a mouse’s lifetime (over 2 years). These long-term studies tend to use either alternate day fasting or restricted single-meal feeding. While these interventions clearly induce torpor on a short-term basis, it is possible that over time mice might adapt to the reduced calorie delivery, adjusting their activity or metabolic rate so that torpor is not required. Hence, the question arises: does torpor continue over prolonged periods of calorie restriction?

In one long-term study of calorie restriction through delivery of a single calorie-restricted meal each day, torpor became increasingly likely over the first month. By the second month, 80–90% of the 40% restriction group and 20–30% of the 30% restriction group were entering torpor each day. This demonstrates that over a prolonged period of restriction (at least up to 3 months), torpor not only continues to occur, but becomes increasingly likely (Mitchell et al. 2015). It seems likely that this pattern continues with more extended periods of calorie restriction, but to our knowledge there have not been any studies that investigate the occurrence of torpor throughout the entire lifespan of mice.

It is likely that the repetitive torpor engagement seen in response to a long-term single-meal feeding paradigm will also be seen under an alternate day fast protocol. Mice under an alternate day fast protocol for up to 19 days continue to engage bouts of torpor on fast days (Swoap et al. 2019), and our data supports this, showing even large adult mice (> 30 g) will enter torpor on consecutive occasions under an alternate day fast protocol (Fig. 3b).

## Could torpor be a confounding factor in studies of dietary restriction?

In the following sub-sections, we explore the fields of aging, cognition, immune function, and behaviour. We discuss whether experiments in each are imposing dietary restrictions sufficient to induce torpor, and if there is evidence from torpor research that engagement of torpor in experimental groups and not control groups would have an effect on outcome measures. Of the studies that use some form of calorie restriction, only one sought to control for torpor whilst implementing caloric restriction, preventing its induction in a group housed at 30°C (Koizumi et al. 1996). The remaining studies neither report any outcome measures that would allow torpor to be identified and quantitated, nor discuss torpor within the manuscript.

### Longevity and general health

Dietary restriction, either through alternate day fasting or provision of less than the ad libitum intake, is widely believed to prolong life-expectancy, as has been discussed in several reviews (Masoro 2000; Speakman and Mitchell 2011; Mattson et al. 2017; Green et al. 2022).

Single-meal feeding with a calorie restriction of between 20 and 40% results in a lifespan extension of 20–40% (Means et al. 1993; Mitchell et al. , 2016, 2019; Pak et al. 2021; Acosta-Rodriguez et al. 2022) and the magnitude of lifespan extension appears directly related to the degree of restriction (Green et al. 2022), just as the degree of restriction impacts the likelihood of mice entering torpor (Mitchell et al. 2015). These restrictions do not need to be implemented from birth to have an effect, with lifespan extensions seen in studies that implement a caloric restriction in adulthood (Weindruch and Walford 1982; Means et al. 1993; Pugh et al. 1999). Improvements in lifespan are typically observed alongside improvements in a range of markers for healthy aging such as reduced expression of genes associated with inflammation (Acosta-Rodriguez et al. 2022), preserved cognitive function (Means et al. 1993), and reduced cancer incidence (Weindruch and Walford 1982; Weindruch et al. 1986; Xie et al. 2017).

The timing of feeding affects the degree of lifespan extension seen in calorie restricted mice just as it affects whether a mouse will enter torpor (Van Der Vinne et al. 2018). Provision of a 30% calorie restriction in a 12-h period (Acosta-Rodriguez et al. 2022) or a single meal (Pak et al. 2021) drives a significant increase in longevity (20–35%). However, the same 30% caloric restricted diet provided across the whole day either conferred a more modest increase (10%) (Acosta-Rodriguez et al. 2022), or no increase (Pak et al. 2021) in lifespan respectively. The authors of the latter study concluded that the lifespan benefits, as well as other health benefits such as reduced kyphosis, improved coat condition, and delayed cancer onset were dependent on a period of fasting (Pak et al. 2021). Similarly, in an alternate day fast paradigm, lifespan extension and delayed cancer onset were still observed despite the mice largely compensating on their re-feed days, such that they experienced only a 7.5% reduction in calorie intake (Xie et al. 2017).

Therefore, single-meal feeding with caloric restriction, which is highly likely to induce torpor, extends lifespan, whilst caloric restriction in which food is delivered evenly throughout the day has a reduced or no effect of lifespan. Hence, calorie restriction is not always sufficient to increase lifespan unless it is delivered in a way that includes a period of fasting. Importantly, this pattern of calorie restriction is also most likely to trigger torpor. There is evidence supporting the hypothesis that torpor plays a role in promoting longevity independently of calorie restriction. Facultative hibernators such as Turkish hamsters enter torpor in response to reduced ambient temperature and shortened photoperiod (Batavia et al. 2013), allowing torpor to be engaged in the absence of calorie restriction. The Turkish hamster in particular lives longer the more time it spends in torpor (with no calorie restriction) (Lyman et al. 1981). Furthermore, metabolic benefits such as improved glucose tolerance and insulin sensitivity are observed in animals that are subjected to fasts with no reduction in total calories consumed (Mitchell et al. 2019; Pak et al. 2021). Therefore, calorie restriction does not appear *necessary* for these health benefits (Lyman et al. 1981). These observations support the hypothesis that torpor engagement might exert an additive effect on the improved lifespan and health of calorie restricted mice and challenge the view that health improvements in calorie restricted groups are purely due to a reduction in total calories eaten (Masoro 2000).

To our knowledge, only one study has actively manipulated torpor engagement whilst measuring the effect of dietary restriction on lifespan extension and lymphoma incidence (Koizumi et al. 1996). In this experiment, C57BL/6 mice on a ~ 50% single-meal fed calorie restriction were housed either at 22°C or 30°C, the latter being too warm for torpor to be observed in mice (Hitrec et al. 2019). There was no difference in survival of the fed control groups at each ambient temperature, and both calorie restricted groups outlived the controls. Critically, the 22°C housed calorie restricted mice had a 41% extension of median lifespan (1143 versus 810 days) and were ~ 33% less likely to die from lymphoma compared to the warm-housed calorie restricted group. These data, alongside the data from experiments that enforce fasts without overall caloric deficit, support the hypothesis that torpor occurrence might be an important factor influencing the outcomes in calorie restriction studies of longevity. Calorie restriction is also associated with extensions in lifespan in a range of homeothermic species where torpor cannot be having an impact. This is discussed later in the manuscript.

### Cognitive function, cognitive decline and neurogenesis

The effects of calorie restriction on cognitive function and memory have also been studied using protocols that are likely to engage torpor. Three months of alternate day fasting increases hippocampal BrdU incorporation (a marker of cell proliferation) and NeuN expression (a marker of cell differentiation) when compared to a diet of just 10% calorie restriction with no fasts (Dias et al. 2021). Torpor bouts would be highly likely to occur during 24-h fast periods (Sunagawa and Takahashi 2016; Hrvatin et al. 2020) (Fig. 2 and 3) but not in response to only a 10% calorie restriction (Mitchell et al. 2015). These changes in hippocampal cell differentiation were associated with improved Morris Water Maze performance in the alternative day fast group (Dias et al. 2021). Similar fasting-dependent changes in both BrdU and NeuN, as well as other markers of neurogenesis and improved memory performance, have been reported in other studies using alternative day fasting (Lee et al. 2002; Li et al. 2013; Baik et al. 2020; Cao et al. 2022). Fasts as short as 12 h can be sufficient to increase expression of markers of hippocampal synaptic strength (specifically, PSD-95) (Baik et al. 2020), when the fast begins at the start of the lights-off period. With that timing, torpor would be likely to be induced (Hrvatin et al. 2020). Therefore, molecular changes associated with learning were enhanced only under dietary restriction conditions that are likely to induce torpor.

There is also evidence that dietary intervention can mitigate age-related decline in cognition. Alternative day fasting protects against age-related declines in BDNF (Brain derived neurotrophic factor – supports neuronal plasticity and growth and is associated with age-related cognitive decline (Budni et al. 2015)) and sirtuin-1 (a protein associated with age-related diseases (Zeng et al. 2009)) expression in senescence-acceleration prone mice (Tajes et al. 2010) and reverses age-related decline in GABAergic synaptic transmission (Bang et al. 2022). A reduction in age-related decline in Morris Water Maze performance is also seen in 30% restriction single-meal protocols (Yang et al. 2014). This behavioural improvement was associated with a reduction in ubiquitinated protein aggregates, and the authors suggested that the calorie restriction protocol improved performance by reducing age-related decline in autophagy (Yang et al. 2014). Both calorie restriction (Halagappa et al. 2007; Rangan et al. 2022) and alternate day fasting (Halagappa et al. 2007; Pan et al. 2022; Elias et al. 2023) appear to protect cognition from deteriorating in Alzheimer’s disease models. These interventions not only slow behavioural decline but additionally appear to drive a reduction in hippocampal Aß (Pan et al. 2022) and phosphorylated tau (Halagappa et al. 2007; Rangan et al. 2022; Elias et al. 2023), both of which are highly associated with Alzheimer’s disease progression (Polanco et al. 2018) and are key targets for therapeutic research (West and Bhugra 2015). Beyond a protective effect, alternate day fasting reverses damage from acute or chronic neurodegeneration in mice (Halagappa et al. 2007; Serger et al. 2022; Yang et al. 2023).

These fasting and calorie restriction results are particularly interesting in light of recent data showing torpor engagement enhances hippocampal long-term potentiation (De Veij Mestdagh et al. 2021) and that torpor appears to protect against diminished Morris Water Maze performance following calorie restriction (Nowakowski et al. 2009). De Veij Mestdagh et al. (2021) used a 2-day fast to induce torpor and found an increase in hippocampal long-term potentiation in the period following arousal from torpor and improved fear learning which lasted up to 4 days. This could represent a mechanism by which daily torpor could, across prolonged periods, support improved memory performance, or confer resilience from age-related cognitive decline.

### Immune function

Immune function is also modified by calorie restriction. Janssen and colleagues (2023) tested the effects of fasting on circulating immune cells in mice (Janssen et al. 2023). A 24-h fast resulted in a significant drop in circulating monocytes, B-cells, T-cells and neutrophils, with the changes observed by 12 h into the fast (Janssen et al. 2023). The timing of the drop in circulating immune cells aligns with when most mice are expected to initiate torpor (Fig. 2). Moreover, exposure to 5 cycles of alternative day fasting doubled survival in mice following an *Escherichia coli* infection (Ganeshan et al. 2019). Single-meal feeding with a 40% caloric restriction also delays the onset of autoimmune disease in susceptible mouse strains (Kubo et al. 1992; Sun et al. 2004), doubling the lifespan of mice in one of these studies (Kubo et al. 1992).

Conversely, 40% calorie restriction reduced survivability from West Nile Virus infection from just under 40% in ad libitum controls to just over 10% in the restricted group, despite calorie restricted mice mitigating thymus involution (Goldberg et al. 2015). Decreased survivability in calorie restricted mice has also been shown in response to polymicrobial sepsis (Sun et al. 2001) and influenza (Gardner 2005). Similarly to the West Nile Virus infection, increased mortality from influenza was observed despite increased splenocyte proliferation (Gardner 2005). Therefore, whilst calorie restriction can lead to molecular changes in immune function, this does not necessarily translate to improved outcomes from infection in-vivo.

Studies exploring changes in immune function due to torpor in mice are limited, but we can draw on evidence from other heterothermic species. Bouma et al (2010) describe the changes in immune function in the 13-lined ground squirrel, reporting no fever response to LPS (lipopolysaccharide – a fever inducing antigen (Kozak et al. 1994)), or PGE1 injection (prostaglandin E1 – a potent pyrogen (Stitt 1973)), and delayed antibody production (Bouma et al. 2010). Circulating leukocytes were reduced up to 90% in torpid 13-lined ground squirrels (Bouma et al. 2010), similar to the drop observed in mice following a 24-h fast (Janssen et al. 2023).

It is very likely that the protocols used in these studies of infection and immune system regulation were sufficiently restrictive to induce torpor, and that this torpor entry will influence infection tolerance at the molecular and organism level, perhaps mediating some of the beneficial or indeed harmful effects seen under different circumstances.

### Motivation for behaviour

Calorie restriction has been used in a range of behavioural tests to assess learning and memory (Ingram et al. 1987; Carlini et al. 2008; Yang et al. 2014; Dias et al. 2021; Cao et al. 2022), anxiety (Wable et al. 2015; Heinz et al. 2021), and anorexia (Chowdhury et al. 2013). Beyond these explicit tests of the impact of caloric restriction on behavioural measures, a caloric restriction is often built into protocols that rely on food rewards to motivate a particular behaviour. Therefore, a broad range of studies are performed on calorie restricted mice, without calorie restriction being the intended independent variable. These studies typically aim to maintain mice at ~ 85% of their ad libitum weight. A 15% reduction in bodyweight was observed under 30% calorie restriction (Ambler et al. 2022), therefore making this a potentially torpor-inducing intervention. Given the additional physical demands of behavioural testing, these caloric restricted mice may even have a higher propensity for torpor than those in our experiments (Schubert et al. 2010).

Precisely when these behavioural tests are administered across the 24-h cycle will impact the state that the animal is in at the time of testing. They could be entering torpor, disturbed from mid-torpor bout or arousing naturally from torpor, and this is likely to affect their ability to meet the physical demands of the test. Currently, relatively little is known about how recent arousal from a torpor bout will affect behaviour. That said, EEG recordings have shown an increase in slow-wave activity following arousal from torpor in the heterothermic Djungarian Hamster (Deboer and Tobler 2003). This change resembles slow-wave activity typically seen following prolonged waking. Whilst there is some debate as to exactly what causes this change in EEG signal, one hypothesis is that deep torpor bouts render animals sleep deprived (Borbély and Tobler 1996; Heller and Ruby 2004). We would expect that sleep deprivation is likely to have an effect, beyond the effects of calorie restriction, on performance in these behavioural tasks.

We anticipate that the majority of these behavioural tests are implemented during the lights-on period. If measurement is performed early in the lights-on period, it is likely that this will coincide with torpor, or arousal from torpor. We therefore encourage researchers to measure and control for torpor induction where possible, and for tests to be applied as late in the lights-on period as possible, or early in the lights-off period to avoid testing animals that have recently aroused from a torpor bout.

## Comparative findings from dietary restriction studies of longevity in homeotherms

We have presented evidence that torpor is indeed likely to be occurring in studies that investigate the effects of fasting or calorie restriction on biological and cognitive processes in mice. As an important potential confound, torpor should be measured and controlled for in experiments moving forwards. However, some of these same calorie restriction protocols have been implemented in ‘strict’ homeothermic mammals that do not enter torpor, and these other species also show health benefits. Studies of the effects of calorie restriction in rats have identified considerable changes in metabolism (Duffy et al. 1989), improved recovery to cervical spinal cord injury (Plunet et al. 2008), improved recovery in a sepsis model (Ma et al. 2022), and extended lifespan (Masoro et al. 1995; Swindell 2012). Interestingly, rats subjected to a 25% calorie restriction only showed enhanced improvements in motor recovery from a T10 thoracic contusion injury when the calorie restriction was combined with an alternate day fast (Jeong et al. 2011). This reflects the evidence in mouse experiments where more restrictive diets promote longer and healthier lives. These data suggest that restrictive feeding provides benefits across species, including those that do not enter torpor, with the benefit relative to the degree of restriction (without malnutrition).

The effects of calorie restriction and fasting protocols on health and longevity have also been explored in humans (Dorling et al. 2020). The degree of the restriction in humans is often less severe than for animals, with an overall calorie restriction of 11.7% achieved in the CALERIE Phase 2 trial (Ravussin et al. 2015), though benefits, such as reduced oxidative stress markers (Il'Yasova et al. 2018) were seen even with these modest restrictions. Calorie restriction in human studies have conflicting results with some fasting protocols causing only small metabolic differences compared with ad libitum fed controls (Heilbronn et al. 2005), and some single-meal feeding protocols leading to poorer glucose tolerance (Carlson et al. 2007) and increased cholesterol and blood pressure (Stote et al. 2007) (it should be noted that in both cases, samples are considerably smaller and diet period considerably shorter than in CALERIE Phase 2). Human calorie restriction studies have yet to assess effects on life expectancy so direct comparisons with the mouse literature is difficult.

Both heterothermic and homeothermic animals experience metabolic and temperature changes in response to calorie restriction (Speakman and Mitchell 2011), and these metabolic changes are associated with a range of other benefits. Nevertheless, torpor is an important physiological factor that is rarely considered in studies of the effects of calorie restriction. Furthermore, we believe that evidence from the field indicates that where torpor is more likely, so the effects of calorie restriction in mice are greater (regardless of the degree of overall calorie restriction). This hypothesis might account for the fact that the benefits of calorie restriction are less striking in primate (Mattison et al. 2012; Cava and Fontana 2013) and human trials than they are in mouse studies.

## Discussion

We have described the feeding protocols used to investigate the effects of dietary restriction on mice. Dietary restriction has largely been studied in relation to its effects on longevity and age-related diseases, however the scope of more recent papers ranges from improved memory and learning to enhanced repair following traumatic nerve damage. Given the range of putative benefits, this is a field of considerable therapeutic interest. We have also laid out the feeding protocols used by researchers explicitly trying to induce torpor in mice. We identify substantial overlap between protocols used by these two fields of research, to the extent that almost all the dietary restriction papers reviewed here are likely to have triggered torpor in many if not all of the experimental mice. We explore how torpor might influence the results of research in longevity, cognitive function, immune function and behaviour, though there are likely more fields being impacted by inadvertent torpor (see recommendations regarding metabolic testing (Ayala et al. 2010)). We do not suggest that the benefits found through research into dietary restriction are entirely due to torpor, particularly given benefits reported in rats and humans—species that do not naturally enter torpor. Rather, we highlight torpor as an extreme metabolic and behavioural change that is vital to consider when performing research in heterothermic species like the mouse, and we advise caution when extrapolating calorie restriction results from mouse studies to species that do not engage in torpor. This review complements two recent studiesexploring the likelihood of dietary restrictions in behavioural experiments inducing torpor (Wilcox et al. 2024) and exploring how torpor bouts can slow epigenetic aging and reduce scores on the mouse clinical frailty index (Jayne et al. 2025). We anticipate the growing interest in torpor research will help to further clarify the fields where torpor might be influencing beneficial outcomes.

We recommend that future studies exploring dietary restriction in mice—either as an intervention in its own right, or as a way to motivate behaviour—consider the possibility of torpor as a factor that could account for some of the outcomes from their caloric restriction, or add unexpected variability in their studies. This may be mitigated by:

- Recording body temperature non-invasively using thermal imaging (Willis et al. 2005; Ambler et al. 2022) (at least in pilot studies) to identify the frequency with which their interventions induce torpor. Use of movement sensors may also detect the occurrence of torpor with prolonged periods of inactivity.
- Considering inclusion of groups with different timing of food administration relative to the start of light / dark period or spreading meals regularly throughout the day (as per (Acosta-Rodriguez et al. 2022)) to systematically increase or reduce the likelihood of torpor induction.
- Conducting experiments in older, heavier mice and at higher ambient temperatures to reduce the probability of torpor induction.
- Consistently reporting the weight change of animals through the experimental period.

We hope that our predictions made in Table 1 are useful for researchers to understand the probability of torpor occurring in their experimental groups, though we note that these predictions might need to be adjusted for differences in genetic strain, ambient temperature and daily physical demands.

## Supplementary Information

Below is the link to the electronic supplementary material.

Supplementary file1: Figure 1 supplemental data - surface temperature (356 KB)

Supplementary file2: Figure 2 supplemental data - Torpor entry data ( XLSX 11 KB)

Supplementary file3 (ZIP 4,474 KB)

## Data Availability

All data supporting the findings of this study are available in the supplementary information.
